# Prenatal Polycyclic Aromatic Hydrocarbon, Adiposity, Peroxisome Proliferator-Activated Receptor (PPAR) γ Methylation in Offspring, Grand-Offspring Mice

**DOI:** 10.1371/journal.pone.0110706

**Published:** 2014-10-27

**Authors:** Zhonghai Yan, Hanjie Zhang, Christina Maher, Emilio Arteaga-Solis, Frances A. Champagne, Licheng Wu, Jacob D. McDonald, Beizhan Yan, Gary J. Schwartz, Rachel L. Miller

**Affiliations:** 1 Division of Pulmonary, Allergy and Critical Care of Medicine, Department of Medicine, College of Physicians and Surgeons, Columbia University, New York, New York, United States of America; 2 Division of Pediatric Pulmonary, Department of Pediatrics, College of Physicians and Surgeons, Columbia University, New York, New York, United States of America; 3 Department of Psychology, Columbia University, New York, New York, United States of America; 4 Departments of Medicine and Neuroscience, Diabetes Research Center, Albert Einstein College of Medicine, Bronx, New York, New York, United States of America; 5 Department of Toxicology, Lovelace Respiratory Research Institute, Albuquerque, New Mexico, United States of America; 6 Lamont-Doherty Earth Observatory, Columbia University, Palisades, New York, United States of America; 7 Department of Environmental Health Sciences, Mailman School of Public Health, Columbia University, New York, New York, United States of America; 8 Division of Pediatric Allergy, Immunology and Rheumatology, Department of Pediatrics, College of Physicians and Surgeons, Columbia University, New York, New York, United States of America; INRA, France

## Abstract

**Rationale:**

Greater levels of prenatal exposure to polycyclic aromatic hydrocarbon (PAH) have been associated with childhood obesity in epidemiological studies. However, the underlying mechanisms are unclear.

**Objectives:**

We hypothesized that prenatal PAH over-exposure during gestation would lead to weight gain and increased fat mass in offspring and grand-offspring mice. Further, we hypothesized that altered adipose gene expression and DNA methylation in genes important to adipocyte differentiation would be affected.

**Materials and Methods:**

Pregnant dams were exposed to a nebulized PAH mixture versus negative control aerosol 5 days a week, for 3 weeks. Body weight was recorded from postnatal day (PND) 21 through PND60. Body composition, adipose cell size, gene expression of peroxisome proliferator-activated receptor (PPAR) γ, CCAAT/enhancer-binding proteins (C/EBP) α, cyclooxygenase (Cox)-2, fatty acid synthase (FAS) and adiponectin, and DNA methylation of PPAR γ, were assayed in both the offspring and grand-offspring adipose tissue.

**Findings:**

Offspring of dams exposed to greater PAH during gestation had increased weight, fat mass, as well as higher gene expression of PPAR γ, C/EBP α, Cox2, FAS and adiponectin and lower DNA methylation of PPAR γ. Similar differences in phenotype and DNA methylation extended through the grand-offspring mice.

**Conclusions:**

Greater prenatal PAH exposure was associated with increased weight, fat mass, adipose gene expression and epigenetic changes in progeny.

## Introduction

Prenatal environmental exposures can increase the risk for several childhood diseases [1], [2]. Among the prenatal exposures of concern are polycyclic aromatic hydrocarbons (PAH) which are a family of air pollutants generated during incomplete combustion and suspected to have both carcinogenetic and endocrine-disrupting properties [3], [4]. PAH are pervasive in the environment and distributed widely in the atmosphere [5]. They can bind to and activate the aryl hydrocarbon receptor (AHR) and other nuclear hormone receptors whereupon they regulate xenobiotic-metabolizing enzymes such as cytochrome P450 [6], [7]. Emerging studies suggest that greater prenatal exposure to PAH is associated with cognitive, behavioral disorders, asthma, and obesity [8]–[12].

Childhood obesity has more than doubled in children and quadrupled in adolescents in the past 30 years. More than one-third of adults and 17% of youth in the United States are obese [13]. An emerging hypothesis is that higher exposure to ubiquitous environmental toxicants may contribute to these childhood growth patterns [14]. For example, in recent epidemiological work from the Columbia Center for Children's Environmental Health (CCCEH), Rundle *et al* found that prenatal PAH exposure, measured by personal ambient air monitoring over a 48 hour period during the 32nd week of pregnancy, was associated with higher body mass index (BMI) and the development of obesity at ages 5 and 7 years [11]. Moreover, in a cross-sectional study using 2001–2006 National Health and Nutrition Examination Survey data, PAH urinary metabolites were associated with higher BMI, waist circumference, and obesity in children and adolescents [12]. Supportive mechanistic studies are limited, but in murine and human adipocyte cells treated with the PAH benzo(a)pyrene, lipolysis was inhibited [15]. *In vivo*, adult males exposed to 15 days of benzo(a)pyrene (0.5 mg·kg^−1^ injected i.p. every 48 h) experienced a 43% gain in weight and an increase in fat mass in the absence of an increase in food intake compared to negative controls [16].

Adipocyte differentiation and function is dependent on several key transcription factors. These include peroxisome proliferator-activated receptor (PPAR) γ, CCAAT/enhancer-binding proteins (C/EBP) γ, and cyclooxygenase (Cox)-2) in both humans and mice [17]–[21]. PPAR γ, which is expressed abundantly in both white adipose tissue (WAT) and brown adipose tissue (BAT), is a nuclear hormone receptor that is necessary and sufficient to promote adipocyte differentiation [22], [23]. C/EBP α, which is expressed late during adipocyte differentiation, works cooperatively with PPAR γ to achieve and maintain the differentiated state of adipocytes [17], [24]. Studies suggest that Cox2 downregulates the expression of the PPAR γ and C/EBP α and therefore inhibits adipogenesis [25], [26]. Overexpression of Cox-2 in WAT also has been shown to increase systemic energy expenditure, induce the development of BAT, and protect mice against high-fat diet–induced adiposity [27]. However, Cox-2 deficient mice have reduced adiposity via reduced production of PPAR γ ligands [19]. Additionally, Cox-2 has been shown to be upregulated following inhalation of diesel exhaust [28], [29]. PPAR γ expression also influences insulin sensitivity, particularly in adipocytes, through its downstream effects [30]. For example, PPAR γ may affect the expression of adiponectin by upregulating its transcription through a PPAR γ-responsive element in the promoter [31], [32] as well as genes pertinent to the etiology to lipogenesis such as fatty acid synthase (FAS) [21], [33], [34].

Prenatal environmental exposures may induce phenotypic changes in offspring through epigenetic modifications and their effects on gene transcription. These may include changes in DNA methylation and histone modifications that affect chromatin packaging [35]. Previously, higher prenatal PAH exposure was associated with increased DNA methylation of interferon γ and acyl-CoA synthetase long-chain family member 3 (ACSL3) in human cord blood; the latter was associated with a greater odds of reported asthma by age 5 years [36], [37]. Moreover, emerging studies suggest that the effects of prenatal environment exposures may extend through the grand-offspring generation. As an example, low dose exposure of dams to the biocide tributyltin (intake of 0.53 and 5.3 µg/kg/day respectively) during gestation increased WAT depots and adipocyte size in offspring (F1), grand-offspring (F2), and great grand-offspring (F3) mice [38]. In another study, dams that ingested approximately 20 µg of the endocrine disrupting chemical (EDC) bisphenol A (BPA) daily during the last 10 days of gestation exhibited differences in social interactions through the F4 mouse offspring [39]. In two epidemiological studies, prenatal smoking was associated with a greater asthma risk in the grandchildren [40], [41]. The support for a role of epigenetic regulation as a mechanism underlying associations between prenatal exposures and alterations in phenotype among grand-offspring so far has been derived from mouse studies. For example, our group showed that prenatal exposure to the allergen *Aspergillus fumigatus* (*A. fumigatus*) decreased DNA methylation at interleukin (IL)-4 CpG^−408^ and CpG^−393^ in grand-offspring mice [42].

We hypothesized that prenatal PAH over-exposure would induce increases in body weight and fat mass in offspring mice. Moreover, we hypothesized that such PAH effects may be associated with altered adipose gene expression and DNA methylation in BAT and WAT. Given our previous report that prenatal *A. fumigatus* administration was associated with changes in CpG methylation in grand-offspring mice [42], we also hypothesized that prenatal PAH would induce increases in body weight and alterations in DNA methylation that extend to the grand-offspring. Our approach was to expose pregnant mice to PAH versus negative control aerosol under relatively physiological conditions via a customized chamber [43], and monitor weight, body fat accumulation, adipose cell size and adipose gene expression and DNA methylation in both the offspring and grand-offspring.

## Materials and Methods

### Animals

**Figure 1:**
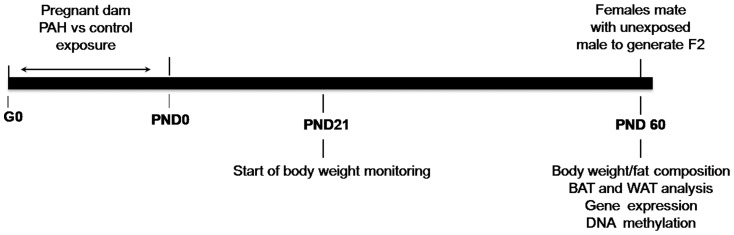
Experiment design. Pregnant female (F0) mice were exposed five hours a day, five days a week throughout gestation days (GD) 0–21 (n = 60 offspring from PAH exposure vs. negative control aerosol, n = 46 dams per group). Beginning PND21, n = 38 in PAH and n = 53 offspring (F1) from 11 litters were weighed every other day. Beginning PND28, total n = 134 mice from 28 litters were weighed every other day. On PND 60, n = 13 female mice from 12 litters in F1 were mated to generate F2 mice. From the remaining mice, n = 21 mice following PAH, and n = 18 mice following negative control were assessed for body fat composition, and fat mass for WAT and BAT at PND60 ±2.46 of age, and gene expression and DNA methylation were measured. Similar outcomes at the same time point (PND60) were measured in F2 mice.

Nine-week-old BALB/cByj female mice (Charles River Laboratories) were housed in a temperature-, humidity- and light-controlled environment and had *ad libitum* access to tap water and breeder chow 5080 (containing 11% fat and 21% protein, Labdiet, St. Louis, MO). Following at least one week period of acclimatization, mice were mated and began the protocol as summarized in Figure 1. Mice were allowed to wean until PND28.

Animal experiments were carried out in strict accordance with the principles and procedures of the Guide for the Care and Use of Laboratory Animals and institutional guidelines. The protocol was approved by the Institutional Animal Care and Use Committee, Columbia University Medical Center.

### PAH Exposure

The PAH mixture was produced by the Lovelace Respiratory Research Institute to replicate the proportions of individual PAH that was measured among a cohort of over 700 pregnant women participating at the CCCEH birth cohort using personal air sampling devices [44], [45], as described in our previous publication [43]. The negative control aerosol solution consisted of 99.97% purified water, 0.02% Tween 80 and 0.01% antifoam (Sigma-Aldrich, St. Louis, MO). The mixed PAH solution was added to yield the final concentration of 7.29 ng/m^3^ (3.69 ng/m^3^ pyrene, plus 3.60 ng/m^3^ from 8 other individual PAH). The 15 ml solutions were delivered via nebulizers (Unomedical Inc., McAllen, Texas) connected to filtered compressed air, as described [43]. Previously we published chamber pyrene (the one chamber PAH measured) levels of 23.24±3.05 ng/m^3^, range 7.38–40 ng/m^3^ from 3 weekly filters extracted together [43], suggesting levels ambient in the chamber may be higher than levels ambient in the NYC urban environment.

The aerosol was delivered beginning on gestational day (GD) 1–3 through GD 19–21 or until day of delivery. Two cages without filter tops were placed in each (PAH, normal air) exposure chamber for five hours a day, five days a week. The exposure chambers were set to achieve a flow of 12.5 to 13.0 liters per minute (LPM). Pressure gauges on the panel were set to 20 psi. At the beginning of each week of the exposure, a 25 mm Pall Flex filter (Pall Life Sciences, Port Washington, NY) with an Amberlite XAD-4 coating combined with a non-coated filter in front to provide support to the brittle XAD coated filter. The filters were replenished weekly and stored in −20°C freezer until analyzed at which time three weekly filters with PAHs were extracted together by methylene chloride (DCM) under sonication. Anhydrous sodium sulfate and flash silica columns were used to purify PAH [46]. Two times of liquid-liquid extractions (DCM and water) were then applied to remove the amphiphilic surfactant, a major component in Tween 80 that was used to emulsify and disperse PAHs in the spray solution. PAH compounds were identified and semi-quantified by 1200 L gas chromatography-tandem mass spectrometer (GC/MS/MS).

### Body weight, fat composition and fat analysis

On PND 0.5, each litter was weighed in total. Birth weight for each dam was calculated as litter weight/litter size. Beginning PND 21, n = 38 offspring (F1) pups from the PAH group and n = 53 from the control group were weighed individually every other day at the same time of the day (middle of the light cycle period). The rest of the mice (n = 134 from 28 litters) were weighed every other day beginning PND28 when the dam was removed from the cage. At PND60, a subset of female and male offspring from each litter was transported to Animal Energy Balance Phenotyping Core of the New York Obesity Research Center at Albert Einstein College. *In vivo* body composition, including total body lean and fat mass using whole body magnetic resonance spectroscopy (MRS) scan for body composition was determined by an investigator blinded to experimental condition [47]. Briefly, mice were placed in a clear plastic tube with a displacement cap to maintain position within the magnetic field of the instrument. 90 second scans were performed on unanesthetized animals. At the end of the studies, interscapular BAT and inguinal, gonadal, and perirenal WAT were extracted and weighed.

### Histologic and morphometric analysis of adipose tissue

A random subset of adipose tissues following over-exposure to PAH (n = 6 male and n = 3 female) and negative control groups (n = 4 male and 4 female) underwent histological analysis as described previously [48]. Briefly, adipose tissues were fixed for 24 h at 4°C in 4% formaldehyde, dehydrated, embedded in paraffin, sectioned (thickness, 3 µm), and stained with hematoxylin and eosin. The tissue slides were scanned and quantified by light microscopy on 5 randomly selected sectional areas per sample (10X, Leica DMR, Wetzlar, Germany). The same region of the fat pad was used for all animals to minimize cell size variation due to differences in anatomical location. The mean diameter of adipocytes was quantified by measuring two randomly selected visual fields in two different sections from each of sample. Approximately 30 cells per mouse sample were averaged using the Image J Software and averaged (version 1.43, NIH, Bethesda, USA). Interobserver agreement in scoring was confirmed by a blinded investigator (κ = 0.878, n = 14 slides for 7 mice, p<0.05). The number of adipocytes seen across standardized visual fields also was counted by two investigators blinded to experimental condition.

### RNA extraction and quantitative reverse transcriptase-PCR (qRT-PCR)

WAT and BAT were dissected from mice at PND60. Total RNA from inguinal WAT and interscapular BAT was extracted using Trizol (Invitrogen) following the manufacturer's protocol. RNA was treated with DNase I (Ambion, Applied Biosystems, Darmstadt, Germany). RNA quality was ascertained using a NanoDrop ND-1000 (Thermo Fisher Scientific Inc., MA, and USA) and the OD A260/A280 ratio calculated to ensure excellent RNA quality. cDNA synthesis was performed with 1 µg of total RNA using SuperScript II and random hexamer primers (Invitrogen). Gene expression assays for PPAR γ, Cox2, C/EBP α, FAS and adiponectin were performed byiQ SYBR Green Supermix (BioRad). The primers used for quantitative RT-PCR are summarized in Table S1 in File S1. Expression of mRNA was measured by quantitative PCR on CFX Connect Real-Time PCR Detection System (BioRad) using the Delta-Delta CT method. Mouse GAPDH transcript served as an internal reference gene.

### DNA quantification and bisulfite conversion

300 mg of interscapular BAT and inguinal WAT were extracted with proteinase K and purified with phenol-chloroform. Genomic DNA was precipitated using sodium acetate/isopropanol followed by washing with 75% ethanol and then resuspended in TE buffer (10 mM Tris-HCl pH 8.0) and stored at −20°C. 250 ng of genomic DNA underwent bisulfite modification utilizing the EZ DNA Methylation-Direct Kit (Zymo Research, Orange, CA, USA).

### Gene-specific DNA methylation analysis

Primers for pyrosequencing were designed with PyroMark Assay Design Software 2.0 (Qiagen) (Table S1 in File S1). PCR was performed according to standard techniques with the following components: 3.0 mM MgCl_2_, 200 µM dNTPs, 0.2 µM primers, 1.25 U HotStar Taq DNA polymerase (Qiagen), and approximately 10 ng of bisulfite-converted DNA per 50 µl reaction. PCR cycling conditions were: 94°C×15 min; 45 cycles of 94°C×30 s, 56°C×30 s and 72°C×30 s; and final extension of 72°C×5 min. Following purification, biotinylated PCR (20 µl) were analyzed using a PyroMark Q24 system (Qiagen). The methylation status of each locus was analyzed individually as a T/C SNP by the PyroMark Assay Design Software 2.0 (Qiagen).

### F2 cohort

At PND60, randomly selected subsets of F1 females per treatment group (n = 9 in PAH and n = 7 in control group, originally from n = 6 F0 PAH and n = 6 F0 control mice, respectively) were mated with adult wildtype unexposed males to generate F2 offspring. Weights began at PND21 for all F2 grand-offspring. Body fat composition and organ fat analysis, gene expression and DNA methylation were measured according to the same protocols.

### Statistical analyses

Body weight, fat composition, gene expression (in triplicate) values are presented as mean ± standard deviation (SD). F1 and F2 body weights were analyzed by two-way ANOVA comparing (group * litter size) for each time point. F2 body weights also were analyzed by two-way analysis of covariance (group * F1 body weight on PND60) for each time point. Physiological and genetic outcomes were compared by Mann Whitney U test at the age of PND60 unless otherwise specified. The relationships between DNA methylation level and mRNA expression fold change and obesity-related phenotypes were determined by Spearman rank correlation coefficients. Comparisons by exposures of weight trajectories were analyzed by generalized estimating equation that controlled for litter size. All analyses were conducted using SAS 9.3 software 64-bit Windows Version (SAS Institute, Cary, NC). Values of p<0.05 were regarded as statistically significant.

## Results

### Greater prenatal PAH exposure was associated with higher weights in F1 offspring

**Figure 2:**
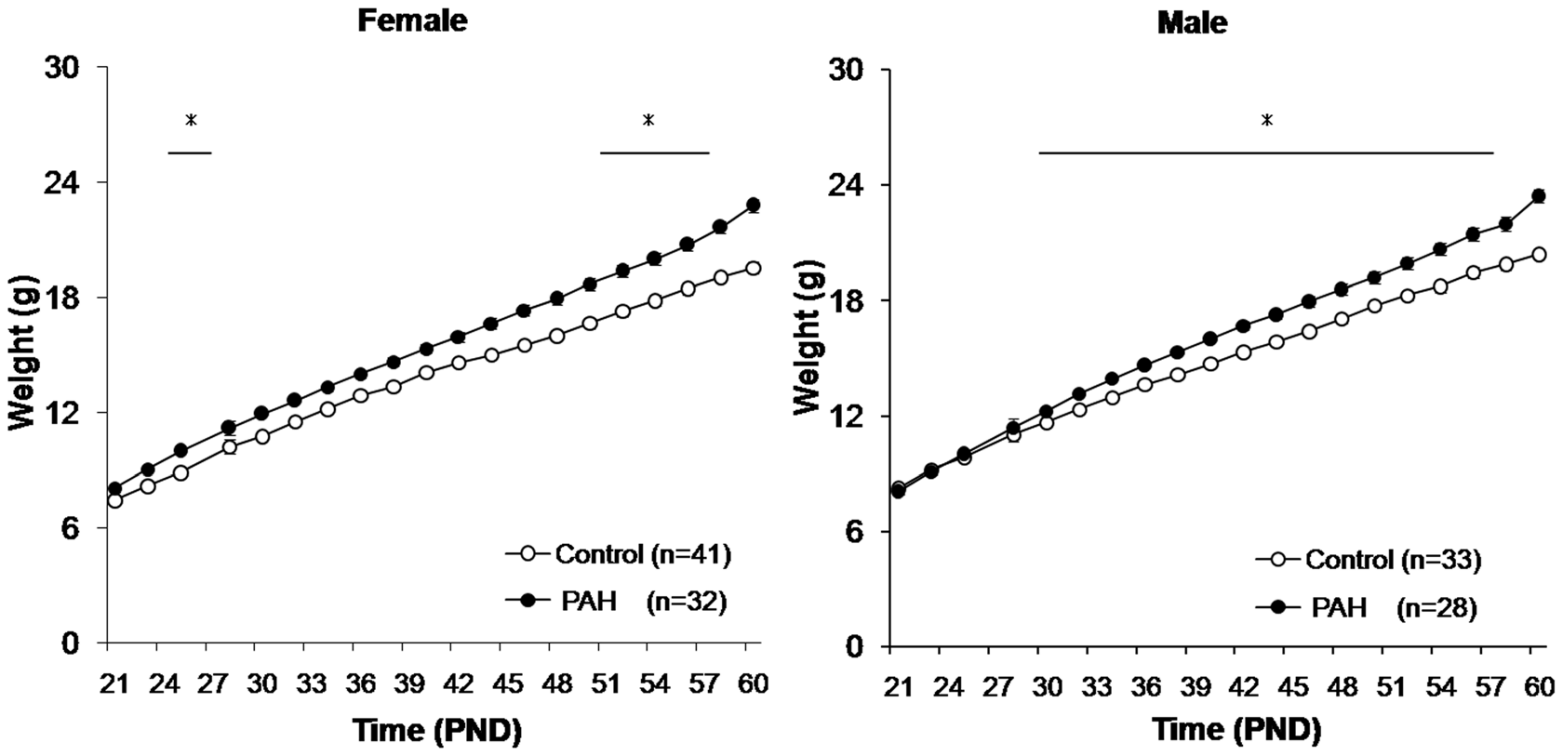
Greater prenatal PAH exposure was associated with greater body weight in male and female F1 offspring. Weights of prenatal PAH and control mice were monitored from PND21 day until PND60. Body weight of PAH and control offspring differed significantly by two way ANOVA (GROUP*LITTER SIZE) on PND 25–27 and PND 52–60 for females and PND 30–60 for males. These included differences among females on PND25 (10.84±0.17 g, n = 19 following PAH vs. 8.87±0.90 g, n = 28 following control exposure), PND27 (10.84±0.17 g, n = 19 following PAH vs. 9.79±0.37 g, n = 28 following control exposure), PND52 (19.36±1.69 g, n = 32 following PAH vs. 17.29±1.79 g, n = 41 following control exposure), and PND 60 (22.79±1.92 g, n = 32 following PAH vs. 19.55±2.05 g, n = 41 following control exposure, p<0.05 for each). Examples of differences among males were found on PND30 (12.24±0.26 g, n = 28 following PAH vs. 11.64±0.35 g, n = 35 following control exposure) and PND60 (23.43±1.85 g, n = 28 in PAH vs 20.38±1.60 g, n = 35 following control exposure, p<0.05 for each). Means ± SD are plotted. *refers to p<0.05 determined at each time point.

Semi-quantitative measures of the pyrene concentrations from the collected filters in the prenatal PAH group averaged 68.4±22.8 ng/m^3^ (range 17.1–168.9) and 19.2±6.4 ng/m^3^ (range 6.8–53.2) in the negative control group. Small and nonsignificant differences in the mean litter size by experimental exposure were observed (4.14±1.20 mice/litter following prenatal PAH vs. 5.28±0.86 mice/litter following negative control exposure, n = 14 litters each, p =  nonsignificant (NS)). The birth weight following prenatal PAH over-exposure did not differ significantly than following the negative control (Table 1). However, using two way ANOVA to account for any effects of litter size on energy metabolism or access to the mother [49], body weight became significantly greater following prenatal PAH exposure than following negative control exposure, evident on PND 25–27 and PND 52–60 for females, and PND 30–60 for males (Figure 2).

**Table 1:** Characteristics of the F1 offspring following prenatal PAH over-exposure versus negative control exposure.

	PAH			Control		
	Male	Female	p-value	Male	Female	p-value
Maternal Exposure (F0)	18	26		18	26	
F1 offspring	28 (47%)	32 (53%)	<0.05	33 (45%)	41 (56%)	<0.05
Litter size	4.14±1.20 mice/litter			5.28±0.86 mice/litter		0.29
Birth weight (g)	1.48±1.89			1.52±1.05		0.88
BMI (g/cm^2^ at PND21)^A^	0.23±0.18			0.22±0.07		0.65
BMI (g/cm^2^ at PND60)^B^	0.53±0.15			0.44±0.34		<0.01

A Based on n = 38 mice following PAH and n = 53 mice following control exposure that began weights this day.

B Based on observation period PND58-PND62.

Birth weight for each dam was calculated litter weight/litter size; body mass index (BMI) (weight/(length)^2^) [63] was calculated for each mouse. Data are presented as mean ± SD. Mann Whitney U test for significance was performed.

### Greater prenatal PAH exposure was associated with greater WAT

Adiposity of offspring also was significantly greater following prenatal PAH when compared to control exposure at PND60, depending on sex of mouse and adipose tissue type. Specifically, the fat mass of inguinal WAT was greater in PAH female (158.10±27.39 mg, n = 10 following PAH vs 124.21±32.16 mg following control exposure, n = 10, p<0.05) but not male (209.37±44.35 mg, n = 8 following PAH vs 160.45±32.49 mg following controls, n = 7, p =  NS, Table 2) offspring. Higher levels in gonadal, but not perirenal, WAT also were observed following PAH over-exposure for both sexes (Table S2 in File S1). In interscapular BAT, differences in fat mass among female and male offspring following prenatal PAH was not evident at PND60 (Table 2).

**Table 2:** Relative fat composition of inguinal white adipose tissue (WAT) and interscapular brown adipose tissue (BAT) at PND60 in the offspring.

F1 Offspring	F- PAH (n = 10)	F- Control (n = 10)	p-value	M- PAH (n = 8)	M- Control (n = 7)	p-value
WAT (mg)	158.10±227.39	124.21±32.16	<0.05	209.25±44.35	160.45±32.49	0.09
BAT (mg)	106.20±30.36	89.08±11.89	0.43	100.37±9.64	93.94±7.01	0.15

Control, offspring following prenatal negative control exposure; PAH, offspring following prenatal PAH exposure.

F-female, M- male.

WAT, BAT tissues were weighed and averaged for each group. The subset of mice was selected at random to across each litter, sex and exposure batch. Data are presented as mean ± SD. Mann Whitney U test for significance was performed.

### Greater prenatal PAH exposure was associated with larger adipocyte size

**Figure 3:**
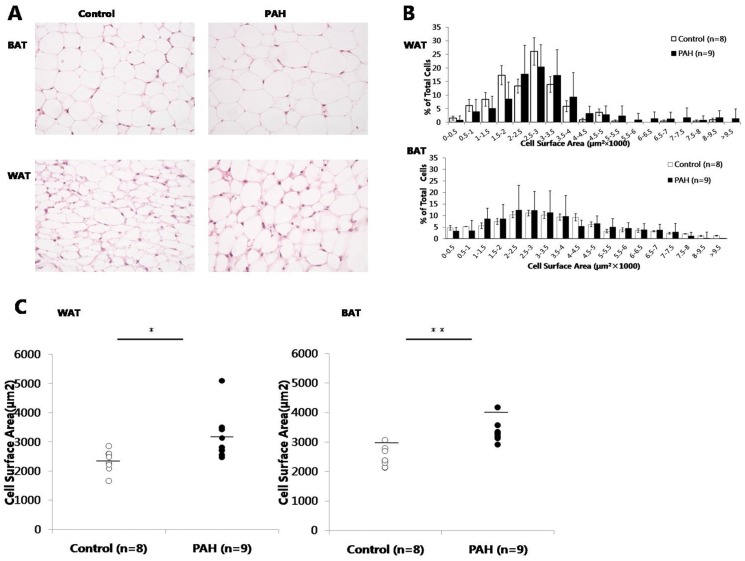
Greater prenatal PAH exposure was associated with increased adipocyte size in the offspring. (A) *Hematoxylin and eosin (H&E) staining of representative of interscapular BAT and inguinal WAT are shown*. (B) *Distribution of adipocyte cell surface area of inguinal WAT (upper panel) and interscapular BAT (bottom panel) following prenatal PAH over-exposure vs control exposure*. A rightward shift is evident following PAH exposure in WAT and BAT. Cross-sectional areas were expressed as mm^2^. Because each millimeter of the digital image equaled ∼50 µm, the calculated areas were multiplied by a conversion factor of 2,500 (50^2^) to determine the cross-sectional area of the adipocytes in µm^2^. Values less than 100 µm^2^ were assumed to represent artifacts from the image-conversion process and were excluded from analysis. (C) *Mean surface area of adipocytes*. Approximately 30 cells were measured for each mouse, averaged and compared between experimental groups. In both inguinal WAT and BAT the mean cell size of PAH was larger than that of control. Mann Whitney U: * p<0.05, ** p<0.01 vs. the control group. M =  male; F =  female.

To investigate whether this increase in inguinal WAT weight among PAH exposed offspring was due to an increase in number and/or size of adipocytes, these parameters were quantified at PND60. Histological examination revealed that over-exposure of pregnant dams to PAH increased the size of adipocyte compared to negative control (Figure 3A). Morphometric analysis demonstrated that there was a shift to the right in the cell size distribution of inguinal WAT adipocyte from prenatal PAH over-exposed mice, and to lesser extent in BAT (Figure 3B). Further, the average adipocyte size in inguinal WAT in the PAH group appeared greater in both female (3,746.08±1232.51 µm^2^, n = 3 following PAH vs. 2,453.96±340.43 µm^2^ following control exposure, n = 4) and male (2,867.26±385.42 µm^2^, n = 6 following PAH vs. 2,238.20±414.90 µm^2^, n = 4, following control) mice. Adipocyte size also was increased in BAT, seemingly in both female (3678.08±441.65 µm^2^, n = 3 following PAH and 2,661.16±392.62 µm^2^ following control exposure, n = 4) and male (3182.64±157.77 µm^2^, n = 6 in PAH vs. 2,279.68±109.24 µm^2^ in control, n = 4). Statistical analyses merged by sex showed significant differences by PAH over-exposure (Figure 3C). Specifically, the average adipocyte size in inguinal WAT in the PAH group was greater than that of control group (3,160.20±815.91 µm^2^, n = 9 following PAH vs. 2,346.08±369.79 µm^2^ following control exposure, n = 8, p<0.05, Figure 3C left panel). Adipocyte size also was increased in BAT (3,347.79±354.51 µm^2^, n = 9 following PAH and 2,477.41±331.29 µm^2^ following control exposure, n = 8, p<0.01). In contrast, differences in adipocyte number across groups by exposure conditions were not found (data not shown).

### Greater prenatal PAH exposure altered PPAR γ, Cox2, C/EBP α, FAS and adiponectin gene expression

Because PPAR γ, Cox 2, C/EBP α, adiponectin, and FAS have been implicated in adipocyte differentiation, insulin sensitivity, and lipogenesis, the effects of prenatal PAH over-exposure on their gene expression in offspring was analyzed. The expression of PPAR γ was up-regulated in the WAT and BAT of offspring following prenatal PAH compared to control mice at PND60 (0.55±0.10 vs. 0.26±0.04 in inguinal WAT, n = 10, p<0.01; and 0.46±0.06 vs. 0.27±0.04 in interscapular BAT tissues, n = 10, p<0.05 in females; 1.04±0.25 vs. 0.40±0.1 in WAT tissues, n = 10, p<0.05; and 0.63±0.18 vs. 0.40±0.11 in interscapular BAT tissues, n =  10, p<0.05 in males, Table 3). Further, there was an increase of Cox2 and C/EBP α expression in the inguinal WAT and interscapular BAT at PND60 among PAH exposed mice when compared to those exposed to the control aerosol (Table 3). Adiponectin and FAS also exhibited altered expression in WAT and BAT, presumably related to a PPAR γ's effects on insulin sensitivity leading to greater adipocyte size and increased lipogenesis (Table 3). These effects were more variable by tissue and sex. FAS levels were increased in female BAT following PAH, but decreased in male WAT and BAT. Adiponectin levels were increased in female BAT and male WAT following PAH over-exposure, but were decreased in male BAT.

**Table 3:** Adiposity gene expression (QRT-PCR) in inguinal WAT and interscapular BAT in offspring.

Tissues	Gene	Female			Male		
		Control (n = 10)	PAH (n = 10)	p-value	Control (n = 10)	PAH (n = 10)	p-value
WAT	PPAR γ	0.26±0.04	0.55±0.10	<0.01	0.40±0.11	1.04±0.24	<0.01
	Cox-2	0.2 4±0.03	0.57±0.08	<0.01	0.38±0.10	1.01±0.23	<0.01
	C/EBP α	0.25±0.03	0.48±0.07	<0.01	0.43±0.12	0.84±0.19	<0.01
	FAS	0.26±0.13	0.18±0.02	0.10	0.29±0.07	0.16±0.01	<0.01
	Adiponectin	0.17±0.12	0.20±0.03	0.14	0.14±0.04	0.33±0.05	<0.01
BAT	PPAR γ	0.27±0.04	0.46±0.06	<0.01	0.40±0.11	0.63±0.18	<0.01
	Cox-2	0.27±0.03	0.45±0.06	<0.01	0.38±0.10	0.55±0.13	<0.05
	C/EBP α	0.28±0.03	0.43±0.06	<0.01	0.43±0.12	0.79±0.18	<0.01
	FAS	0.21±0.02	0.32±0.04	<0.01	0.28±0.07	0.18±0.10	<0.05
	Adiponectin	0.25±0.04	0.51±0.06	<0.01	0.44±0.11	0.29±0.16	<0.05

Control, offspring following prenatal negative control exposure; PAH, offspring following prenatal PAH over-exposure. The mean difference in expression of the target gene was calculated using 2^(−Delta Delta Ct)^. Delta-Delta = Ct_target_- Ct_gapdh_ Data are presented as mean ± SD. Mann Whitney U test for significance was performed.

### Greater prenatal PAH exposure lowered DNA methylation of PPAR γ

**Figure 4:**
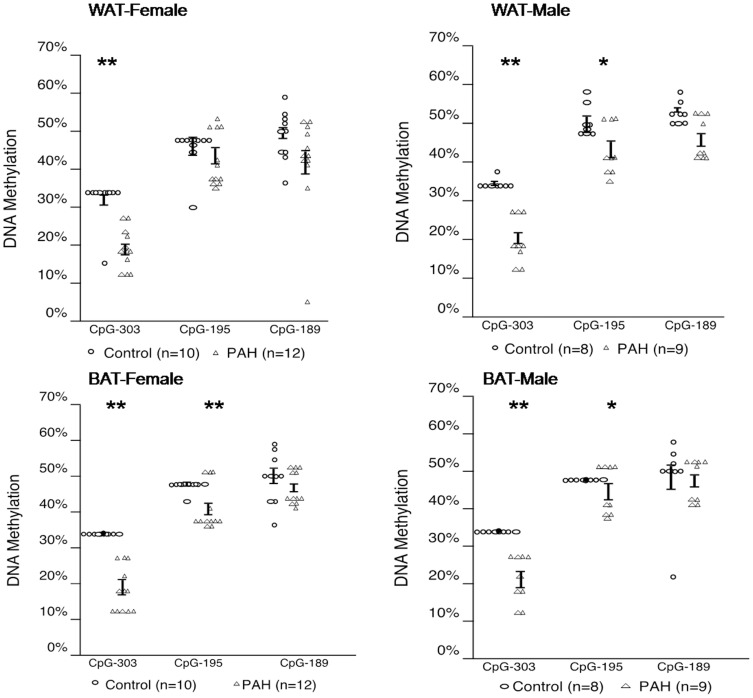
Greater prenatal PAH decreased DNA methylation in PPAR γ promoter at CpG^−303^, CpG^−195^ and CpG^−189^. Data shown were derived from measures in inguinal WAT (n = 12 female and n = 9 male following PAH and n = 10 female and n = 8 male following control exposure) and interscapular BAT (n = 12 female and n = 9 male in PAH and n = 10 female and n = 8 male following control exposure) at PND60. Lines represent ± one SD. Mann Whitney U: * p<0.05, ** p<0.01.

In an effort to determine the epigenetic modifications that accompany the differences in the expression of PPAR γ, DNA methylation of PPAR γ promoter (Figure S1) was analyzed in inguinal WAT and interscapular BAT from prenatally PAH over-exposed and negative control offspring. Differences by over-exposure in methylation varied by site, sex and tissue. For example, differences among female offspring of dams exposed to PAH during pregnancy exhibited in the inguinal WAT were significant at CpG^−303^ (18.9±5.0% in PAH, n = 12 vs. 32.1±6.0% in control, n = 10, p<0.01), but not CpG^−195^ (42.6±6.9% in PAH, n = 12 vs. 44.9±5.3% in control, n = 10, p =  NS or CpG^−189^ (41.8±12.6% following PAH, n = 12 vs. 48.6±6.5% following control, n = 10, p<0.09 (Figure 4, upper left panel). It also was evident that methylation levels, with the exception of one outlier, did not vary by mouse for CpG^−303^ and CpG^−195^ following negative control exposure. However, following PAH over-exposure, more variation in the methylation levels across mice was observed, perhaps consistent with PAH-induced activation of transcription. A similar pattern was detected in the male offspring inguinal WAT at CpG^−303^ (19.8±5.8% in PAH, n = 9 vs. 34.5±1.4% in control, n = 8, p<0.01) and CpG^−189^ (45.9±5.4% in PAH, n = 9 vs. 52.4±2.8% in control, n = 8, p<0.05), but not CpG^−195^ (43.1±6.2% in PAH, n = 9 vs. 50.4±4.2% in control, n = 8, p = 0.09). (Figure 4, upper right panel). In addition, DNA methylation was decreased in interscapular BAT among female mice at CpG^−303^ (18.5±5.9% following PAH over-exposure, n = 12 vs. 34.0±0.9% following control exposure, n = 10, p<0.01) and CpG^−195^ (41.3±5.9% in PAH, n = 12, vs. 46.7±1.0% in control, n = 10, p = 0.05) in female offspring following prenatal PAH over-exposure, but not CpG^−189^ (Figure 4, lower left panel). DNA methylation also was decreased following prenatal PAH over-exposure in interscapular BAT among male mice at CpG^−303^ (21.3±6.0% in PAH, n = 9 vs. 34.0±0.6% in control, n = 8, p<0.01) but not CpG^−195^ or CpG^−189^ (Figure 4, lower right panel).

### PPAR γ DNA methylation levels negatively correlated with inguinal WAT gene expression and obesity-related phenotypes

**Figure 5:**
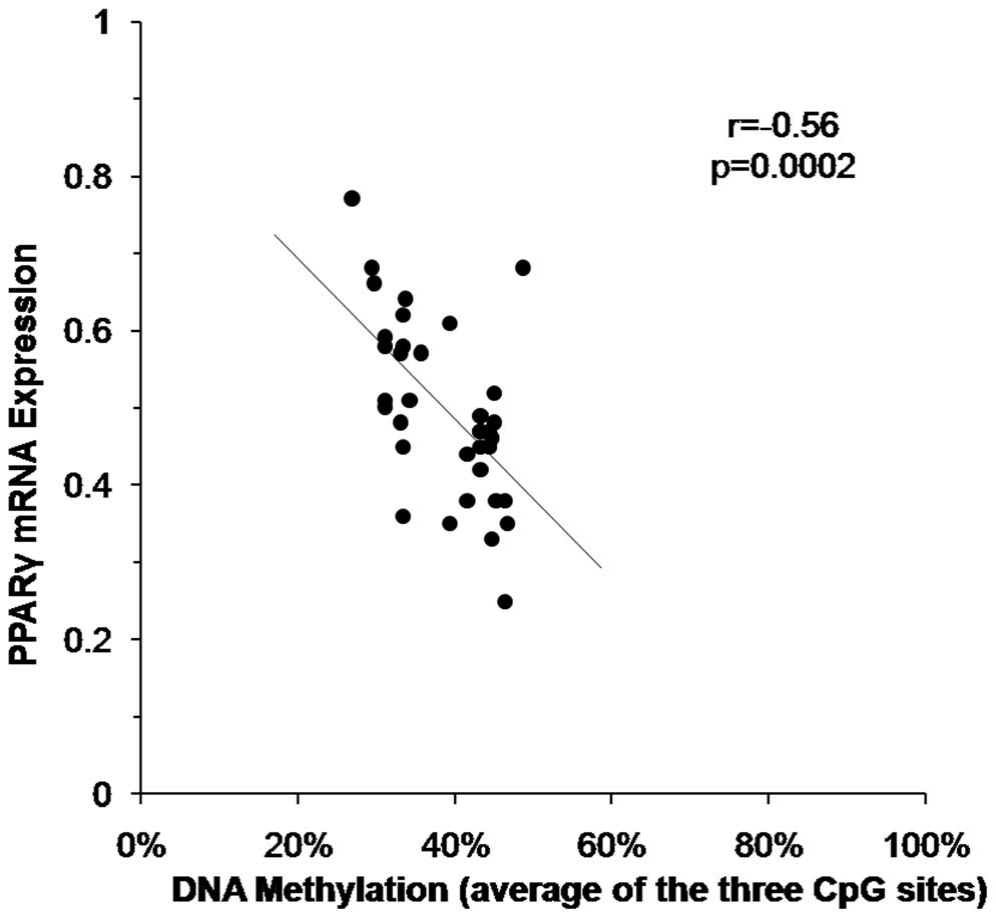
PPAR γ promoter methylation and gene expression in inguinal WAT negatively correlated in F1 offspring. Spearman correlations were conducted on paired PPAR γ promoter methylation (levels average at all 3 sites in promoter) and gene expression levels (r  = −0.56, n = 21 in PAH plus n = 17 in controls, p = 0.002).

A significant inverse correlation between the PPAR γ promoter DNA methylation level and gene expression fold was detected (r  = −0.56, 95% Confidence Interval [CI]  = −0.75, −0.29, p = 0.002) (Figure 5). A similar pattern was detected when comparing individual CpG sites to gene methylation: CpG^−303^ (r = −0.55, 95% CI −0.74, −0.27, p = 0.004), CpG^−195^ (r = −0.33, 95% CI −0.59, −0.01), p = 0.043), and CpG^−189^ (r  = −0.44, 95% CI −0.66, −0.14, p = 0.006, Figure S2A–C). Moreover, the average methylation level of three CpG sites in PPAR γ promoter correlated negatively with body weight and BMI at PND60 (r = −0.43 and −0.62, 95% CI −0.68, −0.17 and 95% CI −0.78, −0.38, p = 0.003 and p = 0.00003, respectively, n = 38) and cell surface area at PND60 (r = −0.61, 95% CI −0.84, −0.18, p = 0.01, n = 17).

### Associations continued through F2 generation

**Figure 6:**
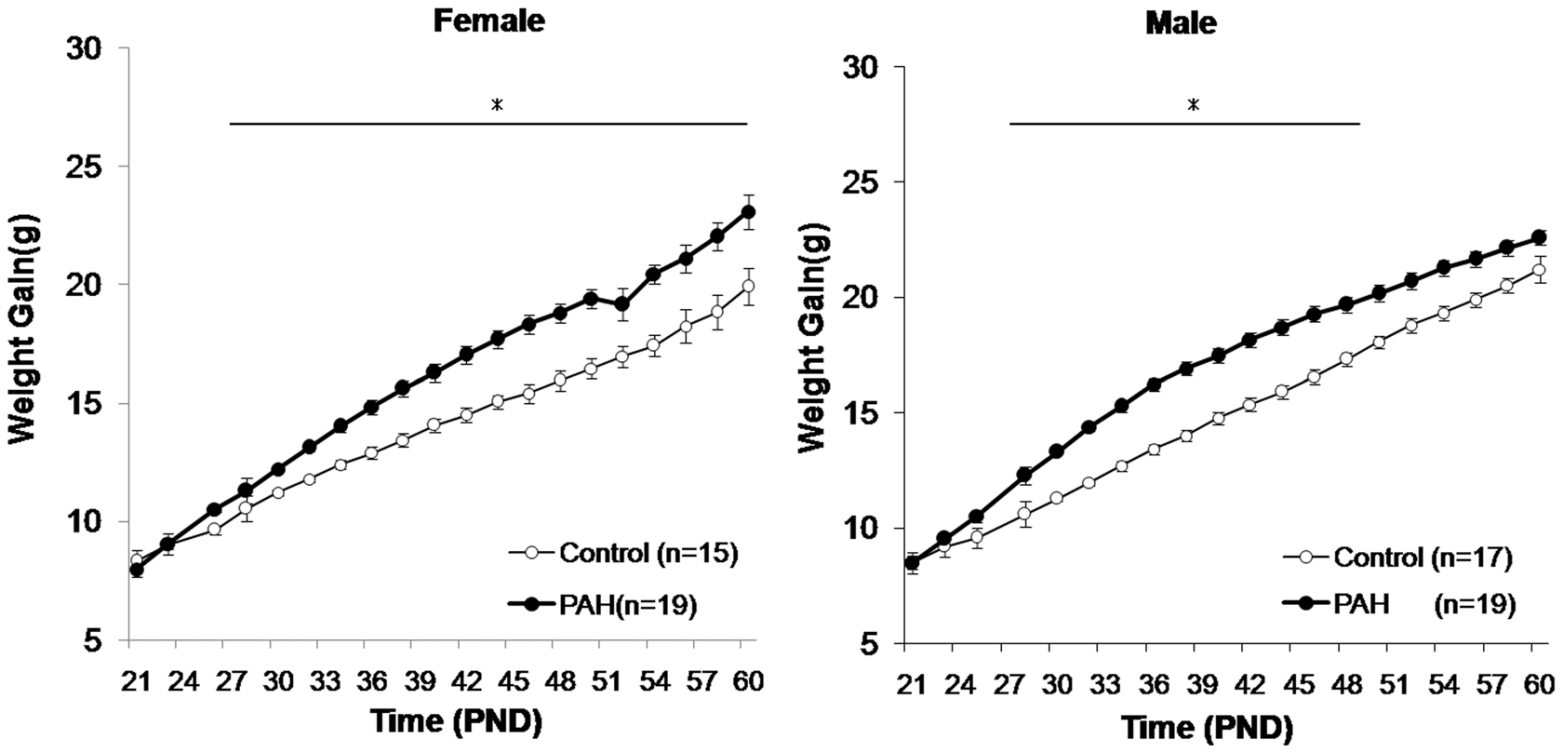
Greater prenatal PAH exposure was associated with greater body weight in F2 male and female grand-offspring. Weights in F2 mice following prenatal PAH versus control exposure were recorded from PND21 through PND60. Left: n = 19 following prenatal PAH, n = 15 following negative control) exposure female mice. Right: n = 17 following PAH and n = 19 following control exposure. Weight following PAH and control exposure among F2 offspring significantly differ by using two way ANOVA (GROUP*LITTER SIZE) for each time points from PND30 to PND60 for females, and from PND27 to PND50 for males. Means ± SD are plotted. *refers to p<0.05 determined at each time point.

In order to determine whether some of these phenotypic and genetic changes continued through the F2 generation, experiments were repeated in n = 36 grand-offspring born to n = 7 F1 dams whose mothers (but not them) were over-exposed to PAH during gestation, and n = 34 F2 grand-offspring born to n = 6 F1 dams whose mothers received only negative control exposure during gestation (in two independent batches of prenatal PAH vs control exposures; Figure 1). Here, small differences in the mean weights of the F2 litters by grandparental over-exposure at birth were detected (Table 4). Further, significant increases in weight following PAH were evident among F2 offspring by using two way ANOVA, adjusting for litter size, for each time point from PND 30 to PND60 for females, and from PND 27 to PND50 for males (Figure 6). The slopes also differed by exposure for both the female (slope beta 0.41 following PAH vs 0.16 following control exposure) and male (slope beta 0.35 following PAH vs 0.31 following control exposure) group of mice (GEE model controlling for litter size, p = 0.0001 for both). In addition, to rule out an effect of maternal obesity, F2 body weights were reanalyzed by two-way analysis of covariance (group * F1 body weight on PND 60) for each time point from PND 21 to PND60, and the female PAH group continued to demonstrate significantly higher body weight from PND38 to PND60, and the males from PND 32 to PND 48 (p<0.05), when compared to controls.

**Table 4:** Characteristics of the F2 offspring following prenatal PAH over-exposure versus negative control exposure.

	PAH		Control		
	Male	Female	Male	Female	p-value
F2 offspring	19 (50%)	19 (50%)	17 (53%)	15 (47%)	0.67
Litter size	5.38±0.61/litter		6.00±0.54/litter		0.28
Birth weight (g)	1.52±0.18		1.63±0.11		<0.01
BMI (g/cm^2^ at PND21)	0.23±0.06		0.21±0.06		0.09
BMI (g/cm^2^ at PND60^A^)	0.51±0.06		0.44±0.06		<0.01

A Based on observing period PND58-PND62.

F2 grand-offspring following prenatal PAH over-exposure were generated from 9 different F1 dams generated from 6 different F0 dams. F2 grand-offspring following control exposure were generated from 7 different F1 dams generated from 6 different F0 dams.

Birth weight in each dam was calculated as litter weight/litter size; body mass index (BMI) (weight/(length)^2^) was calculated for each mouse. Data are presented as mean ± SD. Mann Whitney U test for significance was performed.

**Figure 7:**
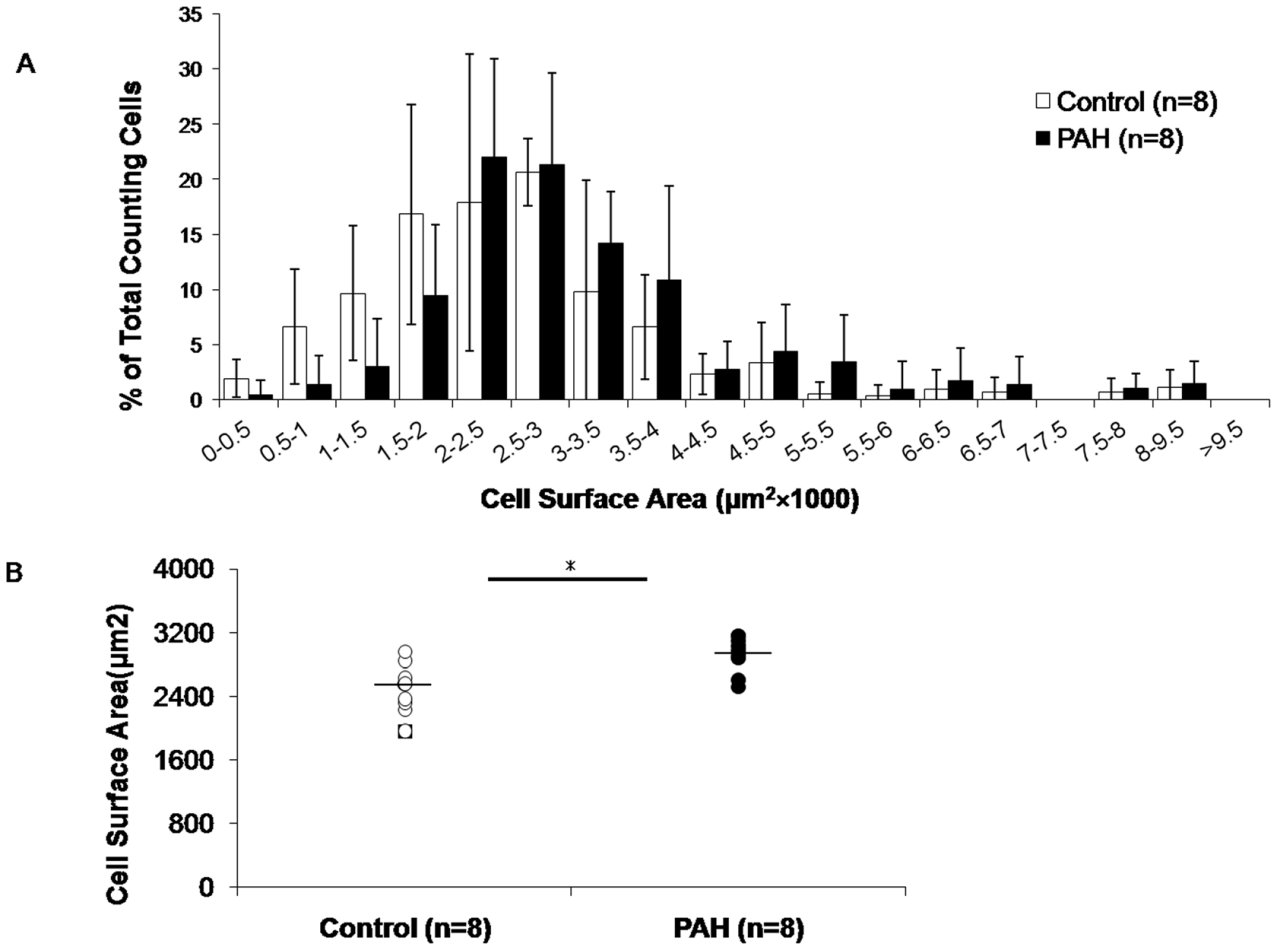
Greater prenatal PAH exposure is associated with increased adipocyte size in F2 grand-offspring. (A). Frequency distribution of adipocyte cell surface area for inguinal WAT following prenatal PAH vs. negative control exposure. (B). Mean surface area of adipocytes of approximately 30 cells were averaged for each grand-offspring mouse at PND60 and compared. Lines represent ± one SD. Mann Whitney U, * p<0.05.

Increases in F2 inguinal WAT following PAH over-exposure were not statistically different for either sex (Table 5). Another nonsignificant association was apparent in two way analysis of covariance that controlled for F1 weight on PND 60 (WAT 134.21±24.00 mg following PAH over-exposure vs. 99.24±23.74 mg following control exposure, p = 0.12). Increases by PAH over-exposure were more apparent in gonadal WAT in F2 female mice and in perirenal WAT in F2 male mice (Table S2 in File S1). In contrast, significant differences between the PAH and control groups in BAT weights were found (female: 64.89 mg ±10.15, following PAH vs. 52.43±6.34 mg, following control exposure, n = 8 each, p<0.05; male: 95.06±20.82 mg, following PAH vs. 77.31±19.50 mg, following control, n = 9 each, p<0.05, Table 5). Similar to the F1 mice, F2 offspring also had a higher percentage of larger adipocytes, and greater mean white adipocyte size, following prenatal PAH over-exposure of F0 mice when compared to control exposure (Figures 7A, B), again without differences in adipocyte number (data not shown). In addition, F2 mice following PAH over-exposure of the F0 dams exhibited greater PPAR γ gene expression in WAT tissues (3.28±1.73 vs. 0.87±0.47 in females, n = 8, p<0.01 and 2.55±0.50 vs. 1.06±0.30 in males, n = 9, p<0.01), as well as Cox2 (1.55±0.82 vs. 0.77±0.60 in females, n = 9, p<0.01 and 0.90±0.23 vs.0.44±0.11 in males, n = 9, p<0.01) and C/EBP α (1.28±0.67 vs. 0.65±0.50 in female, n = 8, p<0.05 and 1.00±0.14 vs. 0.51±0.07 in males, n = 9, p<0.01). Expression of FAS (1.02±0.51 vs. 0.30±0.10 in males, n = 9, p<0.01 and adiponectin (2.74±1.38 vs. 0.8 5±0.16 in males, n = 9, p<0.01) also were increased in male offspring after PAH over-exposure (Table 6).

**Table 5:** Relative fat composition of inguinal WAT and interscapular BAT at PND 60 in F2 grand-offspring.

F1 Offspring	F- PAH (n = 8)	F- Control (n = 8)	p-value	M- PAH (n = 9)	M- Control (n = 9)	p-value
WAT (mg)	135.39±16.21	106.80±23.36	0.37	133.16±20.82	92.51±41.25	0.11
BAT (mg)	64.89±10.15	52.43±6.34	<0.05	95.06±10.82	77.31±19.50	<0.05

Control, offspring following prenatal control exposure; PAH, offspring following prenatal PAH exposure.

F-female, M- male.

WAT and BAT fat were weighed and averaged for each group. A subset of mice was selected at random to across each litter, sex and exposure round. Data are presented as mean ± SD. Mann Whitney U test for significance was performed.

**Table 6:** QRT-PCR for inguinal WAT and interscapular BAT in F2 grand-offspring following prenatal PAH versus negative control.

Tissues	Gene	Female			Male		
		Control (n = 8)	PAH (n = 8)	p-value	Control (n = 9)	PAH (n = 9)	p-value
WAT	PPAR γ	0.87±0.47	3.28±1.73	<0.05	1.06±0.30	2.5 5±0.50	<0.01
	Cox-2	0.77±0.60	1.55±0.82	0.06	0.44±0.11	0.90±0.23	<0.01
	C/EBP α	0.65±0.50	1.28±0.67	0.07	0.51±0.07	1.00±0.14	<0.01
	FAS	0.50±0.25	0.81±0.38	0.14	0.30±0.10	1.02±0.51	<0.01
	Adiponectin	1.06±0.48	1.64±0.77	0.17	0.85±0.16	2.74±1.38	<0.01
BAT	PPAR γ	0.81±0.63	1.27±0.46	0.16	0.91±0.08	2.15±0.18	<0.01
	Cox-2	1.19±0.87	2.64±1.16	<0.05	0.76±0.22	1.61±0.46	<0.05
	C/EBP α	0.59±0.27	2.56±1.82	<0.01	0.61±0.09	1.11±0.30	<0.01
	FAS	0.89±0.15	0.93±0.16	0.37	0.79±0.09	1.12±0.17	<0.01
	Adiponectin	0.99±0.15	1.11±0.14	0.14	0.77±0.10	1.30±0.19	<0.01

The mean difference in expression of the target gene was calculated using 2^(−Delta Delta Ct)^. Delta-Delta = Ct_target_-Ct_gapdh_ Data are presented as mean ± SD. Mann Whitney U test for significance was performed.

**Figure 8:**
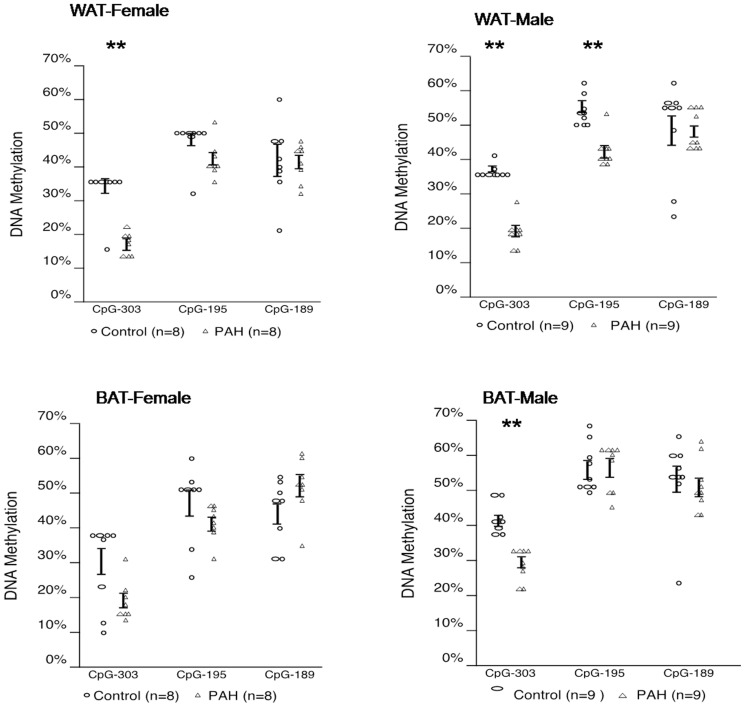
Prenatal PAH decreased DNA methylation in PPAR γ promoter at CpG^−303^, CpG^−195^ and CpG^−189^ in F2 grand-offspring inguinal WAT. Data shown were derived from measures in inguinal WAT (n = 8 female and n = 9 males following PAH or control exposure) and interscapular BAT (n = 8 female and n = 9 male following PAH or control exposure) at PND60. Data are presented as mean ± SD. Mann Whitney U: * p<0.05, ** p<0.01 vs. the control group.

Correspondingly, lower PPAR γ DNA methylation was observed in the inguinal WAT following prenatal PAH among F2 offspring at CpG^−303^ (female: 17.3±3.2% in PAH vs. 33.5±7.1% in control, n = 8 each, p<0.01; male: 18.6±4.1% in PAH vs. 36.7±1.7% in control, n = 9 each, p<0.01) and CpG^−195^ among males (female: 41.9±5.1% in PAH vs. 47.6±6.3% in control, n = 8 each, p = 0.08; male: 42.0±4.6% in PAH, n = 8 vs 53.7±4.2% in control, n = 9, p<0.01). Significant differences in methylation by exposure were not observed at CPG^−189^ in WAT for either sex. Decreased DNA methylation was not apparent in interscapular BAT at CpG^−303^ for female mice (19.1±5.4% following PAH vs. 28.8±11.7% following control, n = 8 each, p = NS), but was for male mice (28.9±4.6% following PAH, n = 9 vs. 41.4±4.2% following control, n = 9, p<0.01). Significant differences in methylation by exposure were not observed at CpG^−195^ or CpG^−189^ in BAT among mice of either sex (Figure 8).

**Figure 9:**
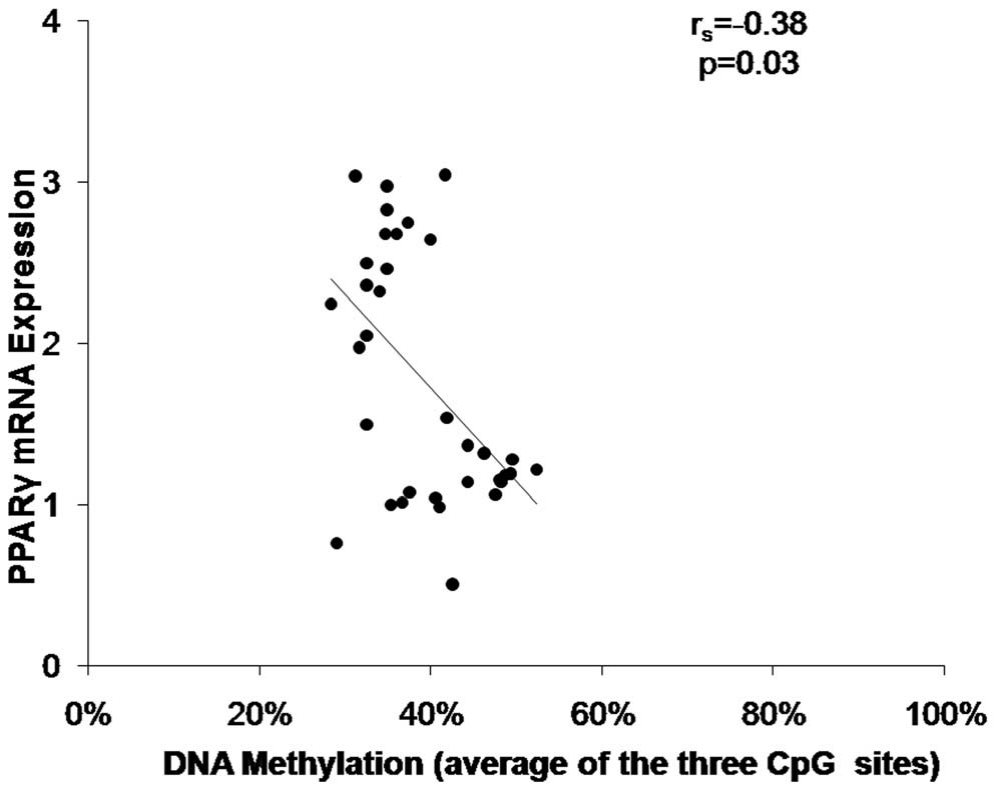
PPAR γ promoter methylation and gene expression in inguinal WAT negatively correlated in F2 grand-offspring. Data shown were derived from measures in inguinal WAT (n = 17 following PAH plus n = 17 following control exposure at PND60). Spearman correlations were conducted on paired PPAR γ promoter methylation (levels averaged at all 3 sites in promoter) and gene expression levels (r_s_ = −0.38, p = 0.03).

A significant inverse correlation between PPAR γ promoter DNA methylation (averaged across the 3 CpG sites) and PPAR γ gene expression in F2 inguinal WAT was apparent (r = −0.38, 95% CI = −0.64, −0.05, p = 0.03 Figure 9). A similar pattern was detected when comparing individual CpG sites to gene methylation at CpG^−303^ (r = −0.54, 95% CI = −0.75, −0.25, p = 0.0008 and CpG^−195^ (r = −0.48, 95% CI = −0.70, −0.17, p = 0.004), but not CpG^−189^ (r = 0.10, 95% CI = −0.25, 0.42). A borderline difference in average methylation by experimental group was apparent in two way analysis of covariance that controlled for F1 weight on PND 60 (35±2.77% following PAH vs 44±5.2% following control exposure, p = 0.065). A significance difference in gene expression by experimental group was apparent in two way analysis of covariance that controlled for F1 weight on PND 60 (2.41±0.50 following PAH vs 1.12±0.23 following control exposure, p<0.0001). The average methylation level of three CpG sites in PPAR γ promoter correlated negatively with body weight and BMI at PND60 (r = −0.61 and −0.51, n = 34, 95% CI = −0.78, −0.34, p = 0.0001 and 95% CI = −0.72, −0.21, p = 0.002, respectively) but not cell surface area (r = −0.21, n = 17, 95% CI = −0.63, 0.30)

### Sensitivity analyses and assessments for batch effects

In order to minimize the possibility of differences in F1 offspring were related to litter size, and therefore access to the dams and amount of suckling rather than prenatal PAH exposure [49], analyses were repeated only among litters of mice of equal size. These were selected per batch of experimental exposure and included 5 litters paired by PAH over-exposure across 4 rounds vs. control exposure. In analyses merged by sex to increase statistical power, we found that PAH over-exposure of pregnant F0 dams was associated with greater body weight on PND42 to PND60 among F1 females, and on PND32 to PND 60 among males (p<0.05, Mann Whitney U). Prenatal PAH remained associated with greater WAT (192±40.9 mg following PAH vs. 143.3 mg±55.6 mg following control exposure, Mann Whitney U, p = 0.024), and greater BMI at PND60 (0.52±0.06 for PAH vs. 0.45±0.03 or control, Mann Whitney U, p<0.0001), and a nonsignificant trend towards higher PPAR γ gene expression (0.58±0.10 for PAH vs. 0.47±0.11 for control, p = 0.06), and lower PPAR γ methylation (36.4±6.3% for PAH vs. 37.8±14.9% for control, p = 0.07, Mann Whitney U for all comparisons).

Finally, to rule out effects related to batch of PAH exposure, differences in phenotype and genotype in F1 offspring (n = 7) and F2 grand-offspring across multiple (n = 2) batches of PAH exposure were examined. Batches did not differ significantly by BMI at PND60, PPAR γ gene expression, or by PPAR γ DNA methylation in the F1 offspring mice. However, F1 body weight on PND60 did differ in one batch compared to the other 6 (p = 0.001). Batches did not differ significantly by body weight, BMI at PND60, PPAR γ gene expression, or by PPAR γ DNA methylation in the F2 grand-offspring mice.

## Discussion

We found that prenatal PAH over-exposure was associated with weight gain and increases in fat mass in offspring and grand-offspring mice. In addition, prenatal PAH was associated with decreased methylation of the PPAR γ promoter and increased expression of PPAR γ, C/EBP α, Cox-2, FAS and adiponectin in adipose tissues.

In NYC and other areas, ambient PAH are ubiquitous [50]. In the US, traffic has been estimated to contribute 46% of the total PAH in the air [51]. Further, obesity remains a serious and growing clinical problem [13]. Childhood obesity clearly has multiple causes such as high dietary fat intake, sedentary behavior and genetic predispositions [52]–[54]. However, prenatal exposure to PAH from traffic emissions and other combustion sources has been implicated as a risk factor for developing childhood obesity in human cohort studies [11], [12]. Here, we were able to identify a novel mechanism that addresses how sustained effects on adipose gene expression can be induced through aerosolized prenatal exposure, and show that such associations extended through the grand-offspring mice. Particular strengths of this study include the relatively physiological and novel system for exposing pregnant mice to aerosolized PAH that mimic individual PAH proportions in urban air previously associated with childhood obesity [11], matching of physiological outcomes with epigenetic ones, and consideration of batch effect and litter size. To our knowledge, this is the first investigation of an association of *in vivo* environmental PAH exposures with later offspring and grand-offspring changes in weight and adipose tissue epigenetic regulation.

Previous cohort studies demonstrated that greater prenatal PAH exposure was associated with decrements in birth weight and length in humans [45], [55], [56]. We found differences in the average birth weight after prenatal PAH over-exposure in the F2, but not F1, grand-offspring. The etiology of this finding is unclear, and could be related to sample size. However, increased weight gain, adiposity, and differences in PPAR γ occurred in studies that controlled for litter size and/or matched by litter size suggesting that these effects were not directly related to greater access of the PAH pups to the dams during weaning that may induce long term effects on energy homeostasis [49].

In contrast to findings at birth, prenatal PAH over-exposure caused a significant gain in weight, apparent by PND 25 among females, and by PND 30 in males, and an increase in fat mass when assessed at PND60. This finding is consistent with Irigaray and colleagues' observations after postnatal exposure to benzo(a)pyrene that also reported resulting increases in fat mass [16], and mirrored the observations by our group and others in urban children [11]. Obesity is characterized by an increase in either the number or size of fat cells [24], [57]. Here we showed that the increased body weight following prenatal PAH appears to be due to increases in fat mass and larger adipocytes in interscapular BAT and inguinal WAT, without apparent increases in the production of new adipocytes. BAT mainly contributes to energy expenditure [58], and its role in the regulation of body weight and development of obesity is less certain, as were the inconsistent associations with PAH exposure. In contrast, WAT seems to be particularly linked to pathological weight gain [59], [60], and type two diabetes [61], and in these studies the association with PAH over-exposure was more robust, especially among the female offspring. There are no published criteria for obesity (or BMI percentiles) in a mouse, and it is difficult to make accurate comparisons of the body weight and other adiposity measures with other mouse models due to differences in genetic backgrounds, age, strain, diet and local environment [62]–[65]. However, the increases in body weight approximating 20% following a high fat diet have been reported elsewhere [66] and are relatively similar to the differences here (Figure 2). In a variety of other exposure models, differences have been similar or greater [27], [63], [67]–[69]. Arguably, our results are comparable to observations made in mouse models following other pollutants. For example, exposure of C57Bl/6J mice to a fine particulate matter aerosol (111.0 µg/m^3^) 6 hours/day, 5 days at week beginning at age 3 weeks caused weight gain and increases in subcutaneous and visceral fat mass by age 12 weeks [70]. Previous studies suggest that sex of the offspring may impact susceptibility to environmentally-induced phenotype [71]–[74]. Differences by sex of mouse offspring were detected in some of our endpoints, including weight gain and its timing and the composition of WAT; however, small sample sizes limited the statistical rigor and effect sizes of these possible differences.

PAH are endocrine disruptor chemicals (EDCs) [75], a class of chemicals associated with childhood obesity [76], [77]. These chemicals can activate PPAR γ, a key regulator in the differentiation of preadipocytes into adipocytes whose activation increases the expression of genes that induce triglyceride uptake and fatty acid storage, and represses genes that induce lipolysis in WAT [78], [79]. Several PAHs also have induced PPAR α and PPAR β/δ activation in human lung adenocarcinoma and HCT-116 colorectal carcinoma cell lines [80]. PPAR γ promoter methylation was reduced following PAH over-exposure, and levels of methylation negatively correlated with gene expression, complementing previous findings that reported the promoter of the PPAR γ gene was demethylated during adipogenesis of 3T3-L1 cells [81]. Its increase in expression here in association with increase in adipocyte size is consistent with findings in PPAR γ knock out mice [20]. Activation of PPAR γ also is known to influence the expression of other genes important to adiposity. C/EBP α, for example, can bind to the C/EBP site in the PPAR γ promoter, providing a regulatory feedback loop [79]. Importantly, the CpG's tested here are located near a C/EBP α binding site (Figure S1). The PAH-induced increase in expression of Cox2 in WAT was not necessarily expected, as its expression has been associated with suppressed PPAR γ and C/EBP α and reduced adiposity [25]–[27]. However, Cox-2 knockout mice exhibited lower body weights and fat, reduced expression of markers of differentiated adipocytes, and reduced production of the PPAR γ activator 15-deoxy-(12,14)-prostaglandin J_2_ [19], suggesting a possible negative feedback related to homeostasis or other mechanisms. In comparison, the PAH induced increased expression of Cox-2 in BAT is more consistent with previous reports of its induction in BAT as part of its role in energy homeostasis and adipose tissue metabolism [27]. Moreover, the effects of prenatal PAH over-exposure on the dysregulation of adiponectin expression are consistent with previous reports in mice following prenatal high fat diet exposure [31], although air pollution-induced effects on this gene as well as FAS have not been described previously to our knowledge.

Also novel is our testing of the physiological and genetic effects of prenatal PAH over-exposure in the grand-offspring. The F1 offspring likely received some direct exposure when the dams inhaled the PAH during pregnancy. While arguably the F2 grand-offspring mice were exposed as germ cells in the F1 offspring, the extension of epigenetic effects to this generation of mice is noteworthy as few studies have examined effects of *in vivo* prenatal exposures on grand-offspring's physiology and DNA methylation. One included our work following prenatal inhaled *A. fumigatus* allergen where altered DNA methylation of the asthma genes IL-4 and interferon-γ were evident in grand-offspring, depending on the timing of exposure during gestation [42]. The greater body weight and steeper trajectory of weight gain, the increased number of large adipocytes, higher body fat mass, adipocyte hypertrophy and the up-regulated expression and the decreased PPAR γ methylation all were observed in the grand-offspring, consistent with sustained effects on key regulatory genes transmitted across lineages.

We acknowledge certain limitations. Prenatal PAH over-exposure likely influenced the expression of additional genes, or methylation of other CpG sites [82]. The results of this study do not indicate whether altered gene methylation occurred prenatally or was due to effects of PAH over-exposure during pregnancy on nutrition, lactation or stress [83], [84]. We were unable to assess gene expression and promoter methylation of PPAR γ at multiple development stages in the offspring to detail better durability of these responses throughout the lifespan of the offspring, nor through additional generations. Even though our wildtype mice on average were heavier, translation to clinical obesity in humans is uncertain where obesity criteria are based on BMI percentiles [85]. Additional tests for food intake, energy expenditure, glucose metabolism and insulin resistance, and serum leptin or lipid profiles in the offspring from prenatal PAH exposure would have been informative [41], [64]. Finally, by exposing only the female dams, patriline effects were not assessed. Exposure of fathers to various toxins, such as cigarette smoke, has been associated with greater BMI in sons [73].

In conclusion, this study provides the first evidence that prenatal PAH over-exposure is associated with the development of adiposity in the offspring and grand-offspring. Persistent changes in body weight accumulation, body fat mass, adipocyte hypertrophy and the upregulated expression in the genes encoding PPAR γ, Cox2, C/EBP α, FAS, and adiponectin are consistent with the development of offspring obesity in these models. Epigenetic mechanisms, at least in PPAR γ, seem to underlie these effects. Potential intervention studies against common urban air pollutants could impact more than one generation.

## Supporting Information

Figure S1 **Location of PPAR γ CpG sites relative to transcription start site (TSS).** Map obtained upon review of TFSEARCH database: http://www.cbrc.jb/research/db/TFSEARCH. Similarly located transcriptional factor binding sites also are shown.
(TIF)

Figure S2 **PPAR γ promoter methylation negatively correlated with gene expression in inguinal WAT.** (A) CpG^−303^ (r = −0.55, p = 0.004), (B) *CpG^−195^* (r = −0.33, p = 0.04) and (C) *CpG^−189^* (r = −0.43, p = 0.006). Delta-Delta Ct values was calculated with Delta Ct experiment- Delta Ct control, and gene expression data of PPAR γ were normalized. n = 21 following PAH over-exposure plus n = 17 following negative control exposure.
(TIF)

File S1 **Tables S1 & S2.** Table S1: Primers sets used for RT-PCR and pyrosequencing. Table S2: Relative fat composition of gonadal and perirenal white adipose tissue (WAT) at PND60 in the offspring and grand-offspring.
(DOCX)
