# Supplementary figures and images for: Prenatal Polycyclic Aromatic Hydrocarbon, Adiposity, Peroxisome Proliferator-Activated Receptor (PPAR) γ Methylation in Offspring, Grand-Offspring Mice
Source: PLoS One. 2014 Oct 27;9(10):e110706. doi: 10.1371/journal.pone.0110706 (PMC4210202; doi:10.1371/journal.pone.0110706)

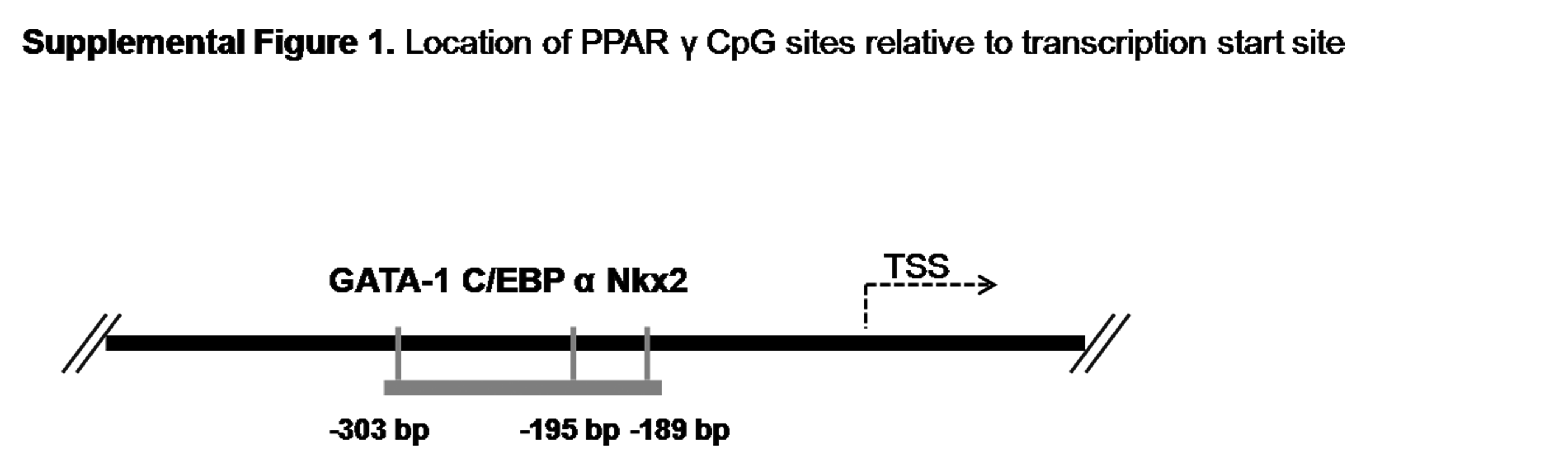

Supplement: Figure S1 — Location of PPAR γ CpG sites relative to transcription start site (TSS). Map obtained upon review of TFSEARCH database: http://www.cbrc.jb/research/db/TFSEARCH. Similarly located transcriptional factor binding sites also are shown. (TIF) [file pone.0110706.s001.tif]

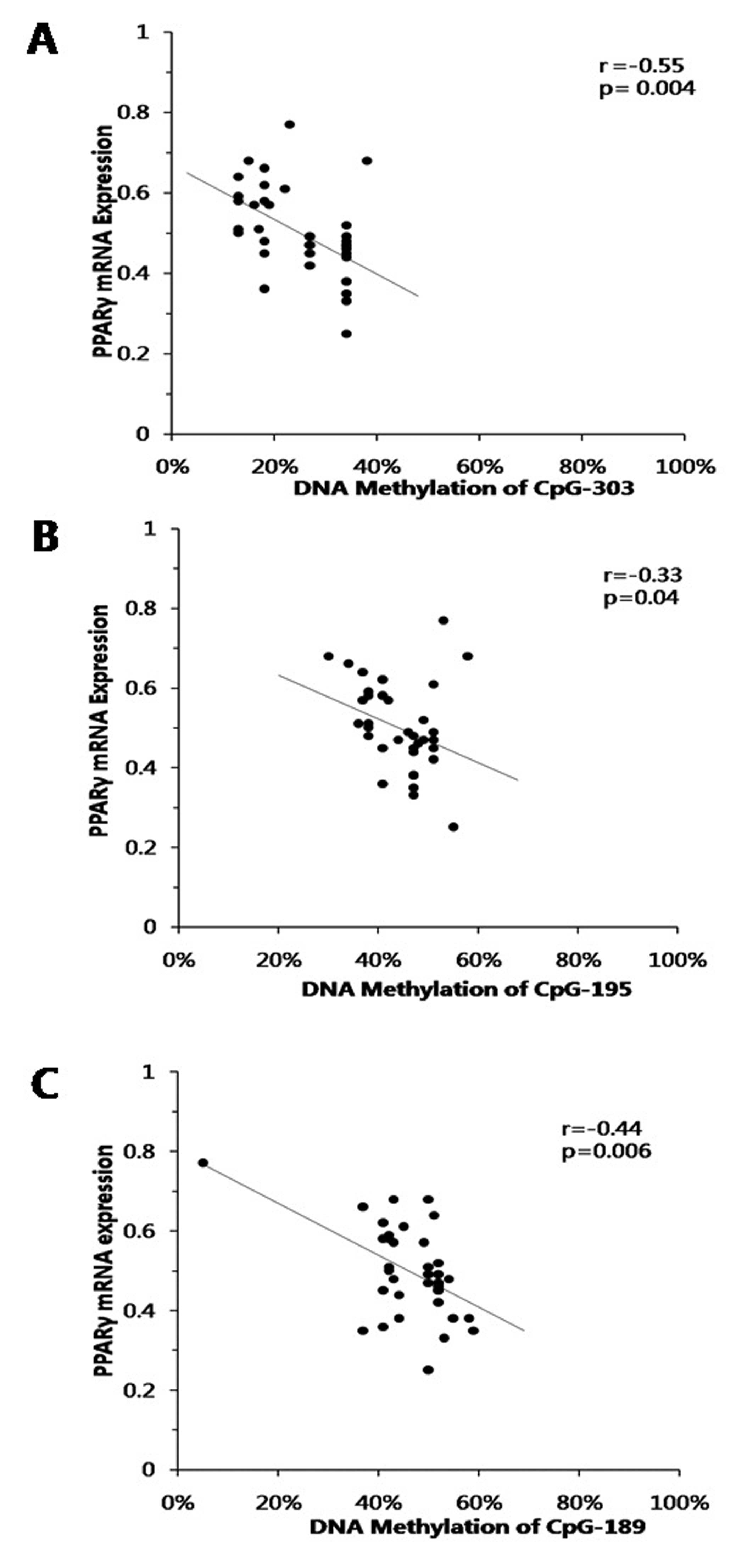

Supplement: Figure S2 — PPAR γ promoter methylation negatively correlated with gene expression in inguinal WAT. (A) CpG−303 (r = −0.55, p = 0.004), (B) CpG−195 (r = −0.33, p = 0.04) and (C) CpG−189 (r = −0.43, p = 0.006). Delta-Delta Ct values was calculated with Delta Ct experiment- Delta Ct control, and gene expression data of PPAR γ were normalized. n = 21 following PAH over-exposure plus n = 17 following negative control exposure. (TIF) [file pone.0110706.s002.tif]
