# Supplementary material for: Prenatal Polycyclic Aromatic Hydrocarbon, Adiposity, Peroxisome Proliferator-Activated Receptor (PPAR) γ Methylation in Offspring, Grand-Offspring Mice
Source: PLoS One. 2014 Oct 27;9(10):e110706. doi: 10.1371/journal.pone.0110706 (PMC4210202; doi:10.1371/journal.pone.0110706)
Supplement: File S1 — Tables S1 & S2. Table S1: Primers sets used for RT-PCR and pyrosequencing. Table S2: Relative fat composition of gonadal and perirenal white adipose tissue (WAT) at PND60 in the offspring and grand-offspring. (DOCX) [file pone.0110706.s003.docx]

Table S1. Primers sets used for RT-PCR and pyrosequencing

| Genes | Primers | Sequence (5’-3’) | Product Size | Application |
| --- | --- | --- | --- | --- |
| PPAR γ | Forward  Reverse | GGAAGACCACTCGCATTCCTT  GTAATCAGCAACCATTGGGTCA | 121bp | RT-PCR |
| Cox2 | Forward  Reverse | TTCCAATCCATGTCAAAACCGT  AGTCCGGGTACAGTCACACTT | 76bp | RT-PCR |
| C/ EBP α | Forward  Reverse | GCGGGAACGCAACAACATC  GTCACTGGTCAACTCCAGCAC | 97bp | RT-PCR |
| FAS | Forward  Reverse | GGAGGTGGTGATAGCCGGTAT  TGGGTAATCCATAGAGCCCAG | 140bp | RT-PCR |
| Adiponectin | Forward  Reverse | GGAGTGTTCGTGGGCTTAGG  GCAGCTCCGGTGATATAGAGG | 140bp | RT-PCR |
| GAPDH | Forward  Reverse | AGGTCGGTGTGAACGGATTTG  GGGGTCGTTGATGGCAACA | 95bp | RT-PCR |
| PPAR γ | Forward  Reverse  Sequencing | GTTGTTTGTTGTTTTGGTAAGATTT  Biotin-ATCTCTACTCTAATAATTCCAACTACTATTTGGTAAGATTTGGTATATT-ATAA | 134bp  122bp | Pyro-sequencing |

Table S2. Relative fat composition of gonadal and perirenal white adipose tissue (WAT) at PND60 in the offspring and grand-offspring

| **F1 Offspring** | **F- PAH**  **(n=10)** | **F- Control**  **(n=10)** | **p-value** | **M- PAH**  **(n=7)** | **M- Control**  **(n=8)** | **p-value** |
| --- | --- | --- | --- | --- | --- | --- |
| Gonadal WAT (mg) | 202.25 ± 47.56 | 147.74 ± 17.01 | <0.05 | 333.96 ± 55.64 | 181.96 ± 95.66 | <0.01 |
| Perirenal WAT (mg) | 92.52 ±  33.58 | 68.96 ±  13.91 | 0.21 | 58.23 ±  9.05 | 46.09 ±  13.04 | 0.06 |
| **F2 Offspring** | **F- PAH**  **(n=8)** | **F- Control**  **(n=8)** | **p-value** | **M- PAH**  **(n=9)** | **M- Control**  **(n=9)** | **p-value** |
| Gonadal WAT (mg) | 160.52 ± 50.71 | 110.12 ± 40.32 | <0.05 | 139.00 ± 53.97 | 97.67 ±  37.59 | 0.10 |
| Perirenal WAT (mg) | 110.38 ± 35.64 | 96.30 ±  14.11 | 0.50 | 148.57 ± 12.99 | 126.96 ± 41.85 | 0.05 |

Control, offspring following prenatal control exposure; PAH, offspring following prenatal PAH over-exposure

F-female, M- male, WAT were weighed and averaged for each group.

Mice were selected at random to across each litter, sex and batch of exposure. Data are presented as mean±SD. Mann Whitney U test for significance was performed.
